# A Speciation Study on the Perturbing Effects of Iron Chelators on the Homeostasis of Essential Metal Ions

**DOI:** 10.1371/journal.pone.0133050

**Published:** 2015-07-20

**Authors:** Guido Crisponi, Valeria Marina Nurchi, Miriam Crespo-Alonso, Gavino Sanna, Maria Antonietta Zoroddu, Giancarla Alberti, Raffaela Biesuz

**Affiliations:** 1 Dipartimento di Scienze Chimiche e Geologiche, University of Cagliari, Cagliari, Italy; 2 Dipartimento di Chimica e Farmacia, University of Sassari, Sassari, Italy; 3 Dipartimento di Chimica, University of Pavia, Pavia, Italy; Northeastern University, UNITED STATES

## Abstract

A number of reports have appeared in literature calling attention to the depletion of essential metal ions during chelation therapy on β-thalassaemia patients. We present a speciation study to determine how the iron chelators used in therapy interfere with the homeostatic equilibria of essential metal ions. This work includes a thorough analysis of the pharmacokinetic properties of the chelating agents currently in clinical use, of the amounts of iron, copper and zinc available in plasma for chelation, and of all the implied complex formation constants. The results of the study show that a significant amount of essential metal ions is complexed whenever the chelating agent concentration exceeds the amount necessary to coordinate all disposable iron —a frequently occurring situation during chelation therapy. On the contrary, copper and zinc do not interfere with iron chelation, except for a possible influence of copper on iron speciation during deferiprone treatment.

## Introduction

Iron chelation therapy is a life-long treatment for blood-transfused β-thalassaemia patients. It was introduced to clinical practice in the 1970s to defend the patients from the effects of iron overload and, in spite of its limits and side effects, it has dramatically improved both life expectancy and quality of life for patients [1]. The first iron chelator to be used in treatment was deferoxamine (DFO): its main drawbacks are the lack of oral activity, its high cost and low compliance. More recently—at the beginning of this century—further work in the field led to the introduction of two additional oral chelators: deferiprone (DFP) and deferasirox (DFX) (S1 Fig).

Any disturbance of the homeostatic equilibria of essential metal ions in the body induced by chelating agents can lead to serious health consequences. A number of reports exist in literature showing the effects of the depletion of essential metal ions during therapy with the three iron chelating agents currently in use. For instance, Al-Refaie et al. [2] reported an “increased urinary zinc excretion in eight patients receiving regular chelation treatment with DFP for up to one year, and subnormal serum zinc values in four, associated with symptoms of dry, itchy, skin patches, which resolved with zinc supplementation in two patients”. In addition, Maclean et al. [3] suggest that cellular zinc content may be a major determinant in iron chelator-induced apoptosis of thymocytes. Galanello [4] also studied zinc levels and found them to be lower in patients treated with DFP compared with controls. A recent review on the safety and efficacy of iron chelation with deferiprone [5] also reports evidence of zinc depletion during therapy.

While investigating the explanation for the nephrotoxic potential of DFX, Hider [6] hypothesizes a possible connection with the formation of zinc polymeric complexes. The author correctly compares ligand affinities of metal chelators on the basis of their pM values, instead of the complex formation constants as many authors still incorrectly do [3]. A recent paper by Erdogan et al. [7] examines the effects of DFX and combined DFO-DFP therapies on serum and urine zinc levels in thalassaemia-major patients. In the serum samples, both therapy cases yielded similar zinc levels—no significant difference between them—that were lower than in control group. Conversely, urine zinc excretion was found to be significantly higher in the treated group than in the controls; further, the DFX group showed zinc levels that were lower than in the group subjected to the combined therapy.

In contrast to zinc, the involvement of copper in iron chelation is not well documented. Pashalidis and Kontoghiorghes [8] suggest, despite the absence of any reports on increased copper excretion during DFO treatment, that the chelation of copper may take place with the redistribution of the chelated metal ions in various body tissues.

Ideally, an iron chelator should possess a sufficiently high selectivity to remove the target iron without interfering with the levels of other metal ions in biological fluids [1]. Therefore, to fully understand its effectiveness, it is important to measure the competition of the various essential metal ions in iron complexation, as well as any perturbations the chelator may induce on the ions’ homeostatic equilibria. These two effects depend on the thermodynamic and kinetic properties of the interaction between the ligand and the involved metal ions [9].

In this work we carefully examine, from a thermodynamic perspective, the complexation of iron(III), zinc(II), and copper(II) by the three ligands employed in clinical treatment of iron overload, under simulated conditions. We present a speciation study that illustrates how the doses of clinical drugs currently used for treatment can affect the homeostatic equilibria of essential metal ions in the body.

## Materials and Methods

### Iron chelating agents used in speciation calculations: deferoxamine, deferiprone and desferasirox

**Fig 1:**
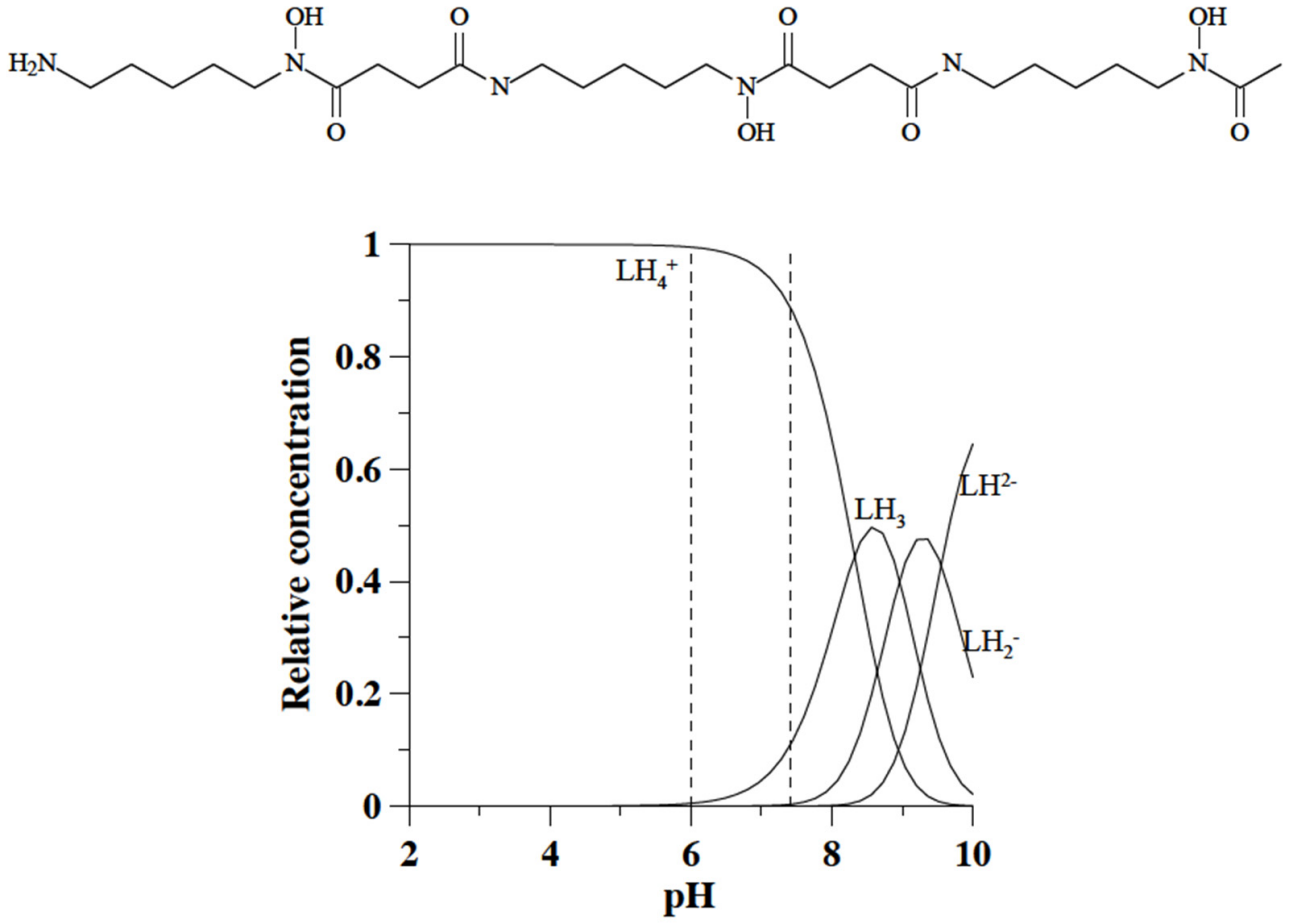
Molecular formula of deferoxamine and speciation plots of its variously protonated forms.

The first chelating agent treated by this study is deferoxamine (DFO), also known as desferal: N'-{5-[Acetyl(hydroxy)amino]pentyl}-N-[5-({4-[(5-aminopentyl)(hydroxy)amino]-4-oxobutanoyl} amino)pentyl]-N-hydroxysuccinamide. DFO was the first drug to be introduced for treatment of iron overload diseases. It is a siderophore produced by *Streptomyces pilosus*, discovered by Prelog and his cooworkers [10]. It is a hexadentate iron chelator, C_25_H_48_N_6_O_8_, with molecular weight 560.68 g/mol, characterized by four protonation constants (log K_1_ = 10.84, log K_2_ = 9.46, log K_3_ = 9.00 and log K_4_ = 8.30 –he first attributed to the terminal amine group, and the other three to hydroxamic groups). At pH 7.4 it is mainly in the fully protonated positively charged LH_4_^+^ form (Fig 1).

Because of its high molecular weight, this drug does not meet the Lipinski criteria [11] for likely orally active molecules. Once it enters the bloodstream it is rapidly eliminated with a half-life of only 5–10 minutes. A small portion of the administered DFO is inactivated within the plasma, while the larger part is uptaken by hepatocytes. The rapid loss of the active compound in circulation explains why prolonged infusion results in more effective chelation [12]. In fact, DFO is usually administered by subcutaneous infusion, 8–12 hours per night, 5–7 nights a week, with a dosage between 20 and 40 mg/kg body weight. More than 60% of excreted iron is expelled through urine; the rest through feces.

**Fig 2:**
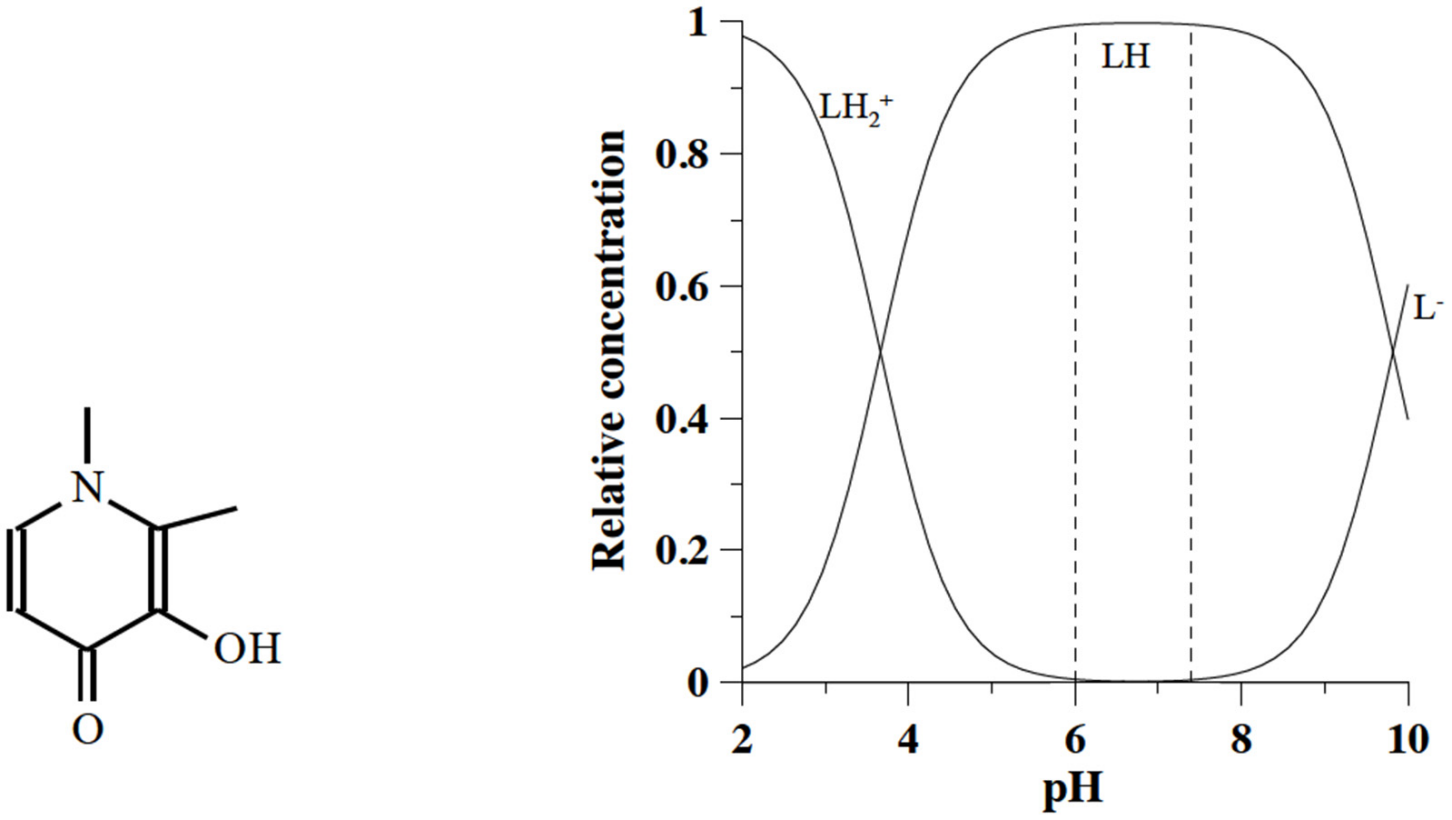
Molecular formula of deferiprone and speciation plots of its variously protonated forms.

Another chelating agent studied in this work is deferiprone (DFP), also known as ferriprox: 3-hydroxy-1,2-dimethylpyridin-4(1H)-one, C_7_H_9_N_2_O_2_, molecular weight 139.152 g/mol. It was originally synthesized by the Hider team at Essex University and patented in 1983 [13]. It is characterized by two protonation constants (log K_1_ = 9.64 and log K_2_ = 3.56), the first related to the hydroxy group and the second to the keto group [14]; at pH 7.4 is found in its neutral form (Fig 2). Thanks to its low molecular weight, it is efficiently absorbed in the intestinal tract. It forms a 3:1 complex with iron and removes iron(III) from ferritin, and even from haemosiderin and transferrin.

The metabolic transformation of DFP in an inactive glucuronide species determines its pharmacokinetic trend. The maximum concentration in plasma is reached two hours after administration, after which it completely declines in the following six hours. This behaviour entails three separate administrations during the day. The majority of the DFP—iron complex formed is excreted by the kidneys (70%). A combined therapy of DFO and DFP turns out to be particularly effective; to reach the same levels of iron excretion with either drug alone, the patient would have to ingest doses so high as to cause serious toxicity effects [15].

**Fig 3:**
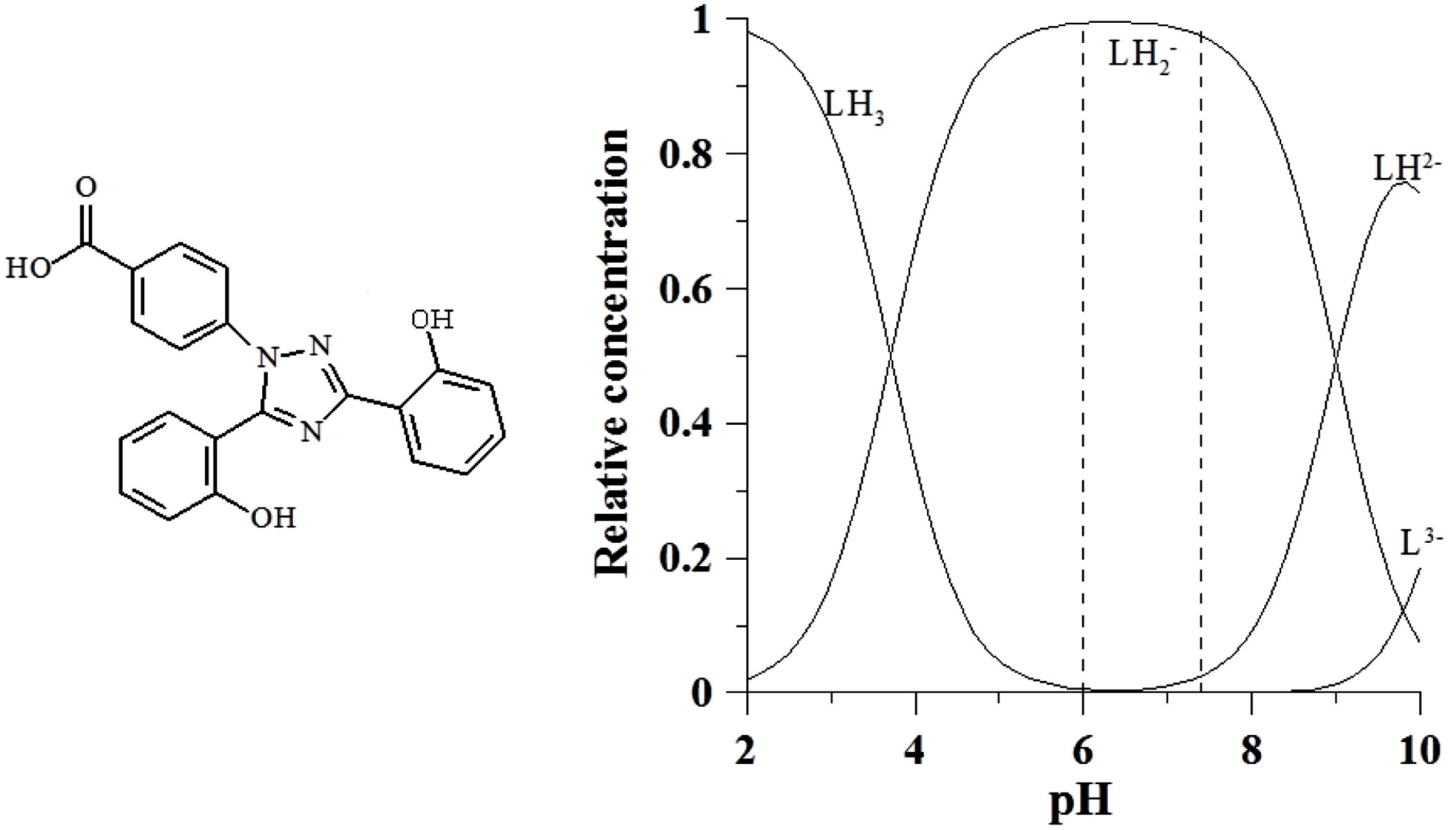
Molecular formula of exjade and speciation plots of its variously protonated forms.

The last therapeutic chelating agent studied in this work is deferasirox (DFX), also known as exjade: 4-[3,5-Bis(2-hydroxyphenyl)-1H-1,2,4-triazol-1-yl]-benzoic acid, C_21_H_15_N_3_O_4_, molecular weight 373.36 g/mol. It was synthesized by Nick et al. at Novartis [16]. It is characterized by three protonation constants log K_1_ = 10.6, log K_2_ = 9.0 and log K_3_ = 3.7, the first two related to the hydroxyl groups and the last one to the carboxylic group. At pH 7.4 it is found in its negatively charged form LH_2_^-^ (Fig 3).

It received EU approval in 2002 and in most other countries in 2006. It is a once-daily oral iron chelator effective in adults and children [17]. At the recommended dose, 20–40 mg/kg/day, its most frequent adverse events include transient gastrointestinal disturbances and skin rash. Renal failure, reported following post-marketing use, is a serious possible side effect, which might be explained by the formation of zinc polymeric complexes in the kidneys [6].

### Speciation

**Fig 4:**
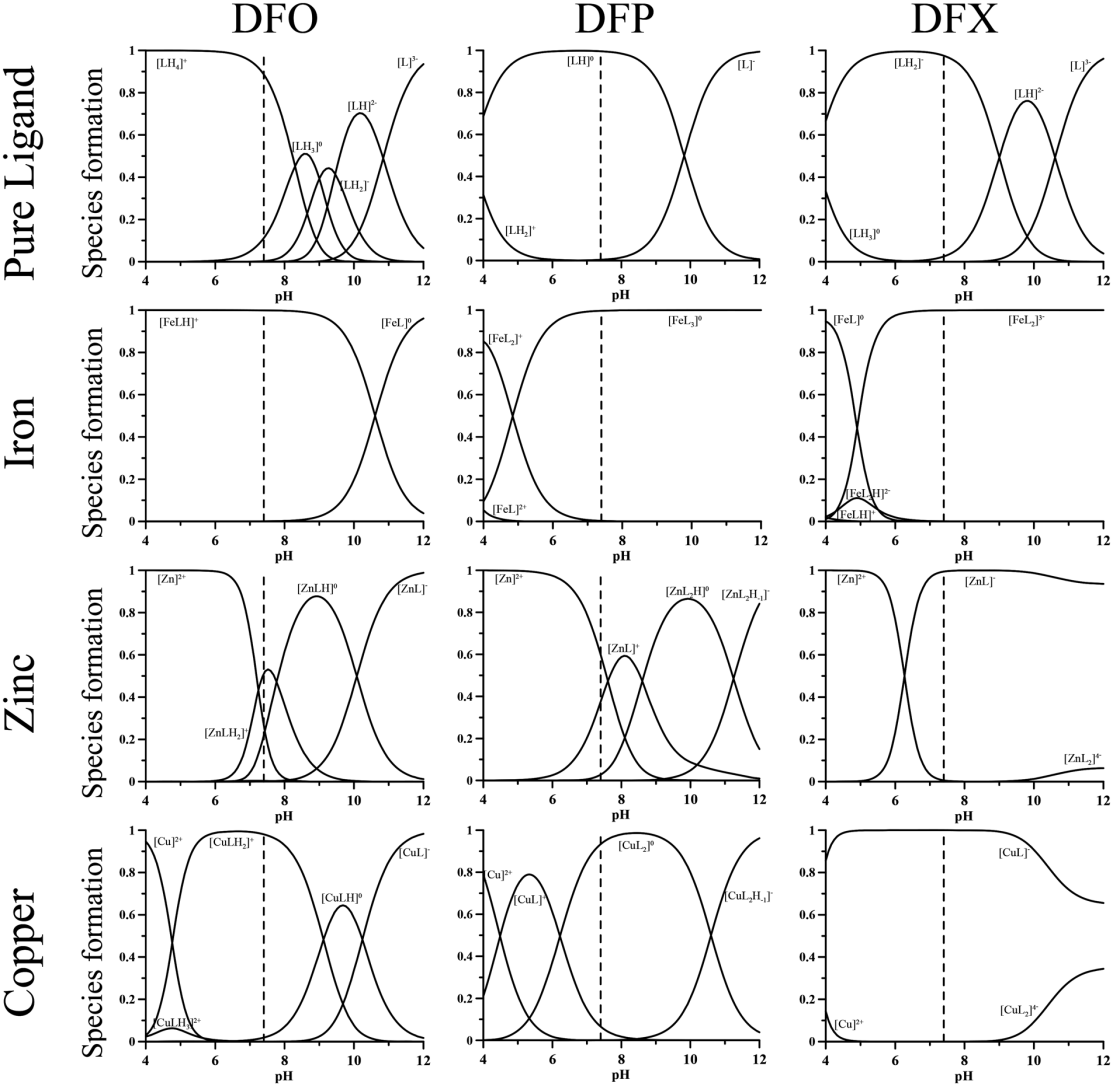
Speciation plots of the three ligands DFO, DFP and DFX and of their iron(III), copper(II) and zinc(II) complexes. Ligand concentration: 10 μmol/L; metal concentration: 1 μmol/L.

All the calculations concerning the speciation were performed with *HYSS Hyperquad simulation and speciation* [18], a software program for the study of equilibria involving soluble and partially insoluble species. The program requires the following inputs: the number of independent chemical species involved in the equilibria (the ligands in their totally deprotonated form, the metal ions and the proton), all the protonation constants of the ligands reported as log β, the stoichiometry of the metal complexes between the metal ions and the ligands, together with the relative complex formation constants (always as log β). Once the inputs are defined, the program, using the option “single point”, allows the calculation of the concentrations of all the species at equilibrium for a given set of total concentrations of ligands and metal ions at a defined pH value. Therefore, to calculate the speciation of our three metal ions of interest in human plasma—the target of chelation, iron(III), and the essential metal ions copper(II) and zinc(II)–in presence of each of the chelating agents DFO, DFP, and DFX, the protonation and complex formation constants of all these ligands with the metal ions of interest have to be known. In addition, the total concentrations in plasma of both metal ions and chelators have to be estimated. The complex formation constants of the three ligands with iron(III), copper(II) and zinc(II), obtained from the IUPAC Database [19] at 25°C and 0.1 M ionic strength, are reported in Table 1 [20–24]. The distribution plots as a function of pH for each metal ion with the three chelating agents are shown in Fig 4, calculated for a metal ion concentration of 1 μmol/L and chelating agent concentration of 10 μmol/L.

**Table 1:** Protonation and complex formation constants for DFO, DFP and DFX with iron(III), copper(II) and zinc(II) from literature.

DFO			DFP			DFX		
Species	log β	pK	Species	log β	pK	Species	log β	pK
[LH]^2-^	^[20]^10.84	10.84	[LH]^0^	^[14]^9.82	9.82	[LH]^2-^	^[21]^10.61	10.61
[LH_2_]^-^	20.3	9.4	[LH_2_]^+^	13.48	3.66	[LH_2_]^-^	19.61	9.00
[LH_3_]^0^	29.3	9.0				[LH_3_]^0^	23.32	3.71
[LH_4_]^+^	37.6	8.3						
[FeLH]^+^	^[22]^41.01	10.61				[FeLH]^+^	^[21, 23]^24.3	2.3
[FeL]^0^	30.4		[FeL]^2+^	^[14]^15.01		[FeL]^0^	22.0	
						[FeL_2_H_2_]^-^	43.4	2.2
						[FeL_2_H]^2-^	41.2	4.3
			[FeL_2_]^+^	27.30		[FeL_2_]^3-^	36.9	
			[FeL_3_]^0^	37.43				
[ZnLH_3_]^2+^	^[20]^33.4	5.2						
[ZnLH_2_]^+^	28.17	7.77						
[ZnLH]^0^	20.4	10.1						
[ZnL]^-^	10.32		[ZnL]^+^	^[24]^ 7.24		[ZnL]^-^	^[21, 23]^12.1	
						[ZnL_2_]^-4^	16	
			[ZnL_2_]^0^	13.55	11.25			
			[ZnL_2_H_-1_]^-1^	2.30				
			[ZnL_3_]^-1^	15.2				
[CuLH_3_]^2+^	^[20]^36.99	3.89						
[CuLH_2_]^+^	33.1	9.1						
[CuLH]^0^	23.98	10.24						
[CuL]^-^	13.74		[CuL]^+^	^[14]^10.42		[CuL]^-^	^[21, 23]^17.6	
						[CuL_2_]^-4^	22.4	
			[CuL_2_H]^+^	21.98	2.89			
			[CuL_2_]^0^	19.09	10.60			
			[CuL_2_H_-1_]^-1^	8.49				
[Cu_2_LH]^2+^	32.09							

[FeLH]^+^, [ZnLH_2_]^+^, [ZnLH], [CuLH_2_]^+^ are the main species with DFO identified on the speciation plots in Fig 4 at pH 7.4. In [FeLH]^+^ iron is coordinated by the three hydroxamate groups, being the terminal amine nitrogen atom still protonated. In [ZnLH_2_]^+^ and [CuLH_2_]^+^ the divalent metal ions are coordinated by two hydroxamate groups, being the third and the amino groups still protonated. In the neutral [ZnLH] zinc is coordinated by all three hydroxamate groups.

Above the physiological pH value, the calculation of pK_MLH2_^+^ (M indicates whichever metal) for the protonated complexes reveals that the pK value of zinc(II) is distinctly lower than that of the free ligand, but it is comparable in the case of copper(II). This indicates that zinc(II) ion is able to coordinate all three hydroxamate groups in [ZnLH] and in [ZnL]^-^, whereas only two of them are bound to the copper(II) in the mononuclear species [CuLH] and [CuL]^-^, and the third hydroxamic group binds a further copper ion giving the dinuclear complex [Cu_2_LH]^2+^ in presence of copper excess [20].

With DFP at pH 7.4, iron is totally found as the neutral species [FeL_3_], zinc is only partially chelated—being that [ZnL]^+^ and [ZnL_2_] are present at about 45% and 3%, respectively—and copper exists as the neutral species [CuL_2_], with only negligible amounts of [CuL]^+^.

With DFX, the iron(III), zinc(II), and copper(II) ions are totally complexed at pH 7.4; iron(III) as [FeL_2_]^3-^, while the bivalent metal ions as [ZnL]^-^ and [CuL]^-^ species. On the other hand, with DFO and DFP the selectivity of iron(III) with respect to the divalent metal ions depends on the presence of coordinating groups composed exclusively of hard oxygen atoms. In the case of DFX, which has two oxygen and one nitrogen coordinating atoms, the selectivity is a consequence of its particular structure that allows it to obtain a stable complex only with small ions such as iron and aluminium [21].

### Estimate of the concentration of metal ions in human plasma

#### Iron

According to “Guidelines for the Management of Transfusion dependent Thalassaemia” [25], thalassaemia-major patients receive 100–200 mL of pure red blood cells per Kg of body weight per year, equivalent to 116–232 mg of iron/Kg body weight/year, or 0.32–0.64 mg/Kg/day. In addition, a dietary iron absorption of about 1–2 mg/day has to be considered, contributing between 2% to 10% to the total iron income. A proper chelating strategy has to reach a stationary equilibrium between loaded and excreted iron. The necessary amount of each chelating agent to chelate 1 mg of iron can be calculated, depending on both the ligand’s molecular weight and the stoichiometry of the formed complex. Actually, a mole of iron is chelated by 1 mole of DFO, 2 of DFX and 3 of DFP, so that 1 mg of iron needs 11.8 mg of DFO, 13.4 of DFX and 7.5 of DFP. The suggested doses for clinical use are reported in Table 2.

**Table 2:** Daily doses of iron chelators.

Iron chelator	Dose (mg/Kg/day)	Administration
DFO	20–40	subcutaneous infusion for 8–12 hours
DFP	75–99	1/3 of a dose three times a day
DFX	20–40	once daily

The doses, compared to the necessary stoichiometric amounts, are 5 times greater for DFO, 7–10 for DFP, and 2.5 for DFX. Taking into account these data and using some simplifying assumptions, the concentration of iron and of chelating agents in human plasma can be estimated. A reference man [26] weighting 70 Kg, with 5.2 L of circulating plasma is assumed as a model. Disregarding the dietary iron intake, transfusional iron amount ranges from 22.4 to 44.8 mg/day. Let us assume that this quantity is released in 5.2 L of circulating plasma in 24 smaller doses, one each hour, and that a stationary state is reached between released iron and excreted chelated iron. We also assume that transfusional iron in body compartments other than plasma is negligible, which is equivalent to considering the iron in all compartments accessible by iron chelators in the same way as that circulating in plasma. At these conditions, we can assume a concentration of circulating iron in plasma between 3.2 μmol/L and 6.4 μmol/L, with a rounded mean value of 5 μmol/L. This amount of iron circulating in the plasma of transfused thalassemia patients is mostly bound to completely saturated transferrin, and the excess to endogenous ligands such as albumin and citrate, with a distribution pattern that depends on the specific conditions (amount of iron, relative concentrations of endogenous ligands, etc.) [27, 28]. The large number of factors affecting the level of circulating iron, its speciation and its exchange ability makes it exceedingly difficult to estimate the concentration more precisely—and futile, since the assumed 5mmol/L concentration is adequate to reliably describe the action of chelating agents on essential and target metal ions.

#### Zinc and copper

The literature on zinc(II) in human plasma agrees well with a total concentration of about 12 μmol/L [26–32], and on the fact that about 70% is found bound to albumin while the rest is bound to α_2_-macroglobulin. The total zinc concentration can be almost doubled if the daily amount of excreted zinc after CaNa_2_EDTA or 2,3-dimercapto-1-propanesulfonic acid treatment is taken into account [33–35]. In speciation calculations the 12 μmol/L of zinc can not be considered freely chelatable by iron chelators, and the contemporary equilibria with albumin must also be considered. The binding constants for the zinc-albumin interaction are scattered in the literature, expressed in different units and without establishing the effect of pH variations. Nevertheless, all authors seem to agree that the imidazole groups are responsible for zinc binding to albumin. There are 16 imidazole groups in albumin, so we assume that in the calculation the binding to 16 imidazole ligands can substitute the binding to one albumin molecule. We performed a simulation with HYSS using the complex formation constant for a 1:1 complex imidazole-zinc, assuming a total zinc concentration 12 μmol/L and a total imidazole 16 x 6.76 x 10^−4^ mol/L = 0.0108 mol/L– 16 imidazole ligands per album molecule and 6.76 x 10^−4^ mol/L being the concentration of albumin in human plasma. The calculated concentration of complexed zinc at pH 7.4 corresponds to 73% of the total zinc, confirming the fidelity of the simulation.

Using this simulation, the ternary system “albumin”-iron chelator-zinc was studied using a zinc(II) concentration 12 μmol/L, imidazole 0.0108 mol/L and DFO 20 μmol/L, DFP 15–70 μmol/L and DFX 20–80 μmol/L. The results reported in Table 3 show that all the three chelating agents are able to extract metal ions from albumin in different amounts.

**Table 3:** Speciation study of the systems “Albumin”-iron chelator-metal iron. Concentration of the complexes formed between metal ions and iron chelators in presence of albumin, expressed as μmol/L. C1 and C2 represent the complexes MLH_2_ and MLH with DFO, and ML and ML_2_ with DFP and DFX.

	L	C1	C2	MAlb	M_free_
DFO-Zinc	20	4.37	1.87	4.19	1.53
DFO-Copper	20	11.76	0.22	—	—
DFP-Zinc	70	4.99	2.33	3.43	1.26
	15	2.13	0.20	7.07	2.59
DFP-Copper	70	0.14	11.80	—	—
	15	3.00	5.48	3.48	—
DFX-Zinc	80	11.96	—	0.03	0.01
	20	11.72	—	0.20	0.07
DFX-Copper	80	11.99	—	—	—
	20	11.99	—	—	—

According to Linder et al. [36], about 6 mg of copper circulates in plasma bound to various endogenous transport proteins other than albumin, corresponding to a 15 μmol/L total copper concentration. In the following the copper concentration is assumed to be ranging from 2 to 5 μmol/L on the basis of excreted copper after treatment with CaNa_2_EDTA [33], considering only the non-ceruloplasmin-bound exchangeable copper in plasma [37] and taking into account the albumin interaction. In our opinion in such situations the distribution of the metal ions among different species is not only dictated by the thermodynamic properties of the system, but by the kinetic ones too [38].

### Estimate of the concentration of chelating agents in human plasma

#### DFO

**Fig 5:**
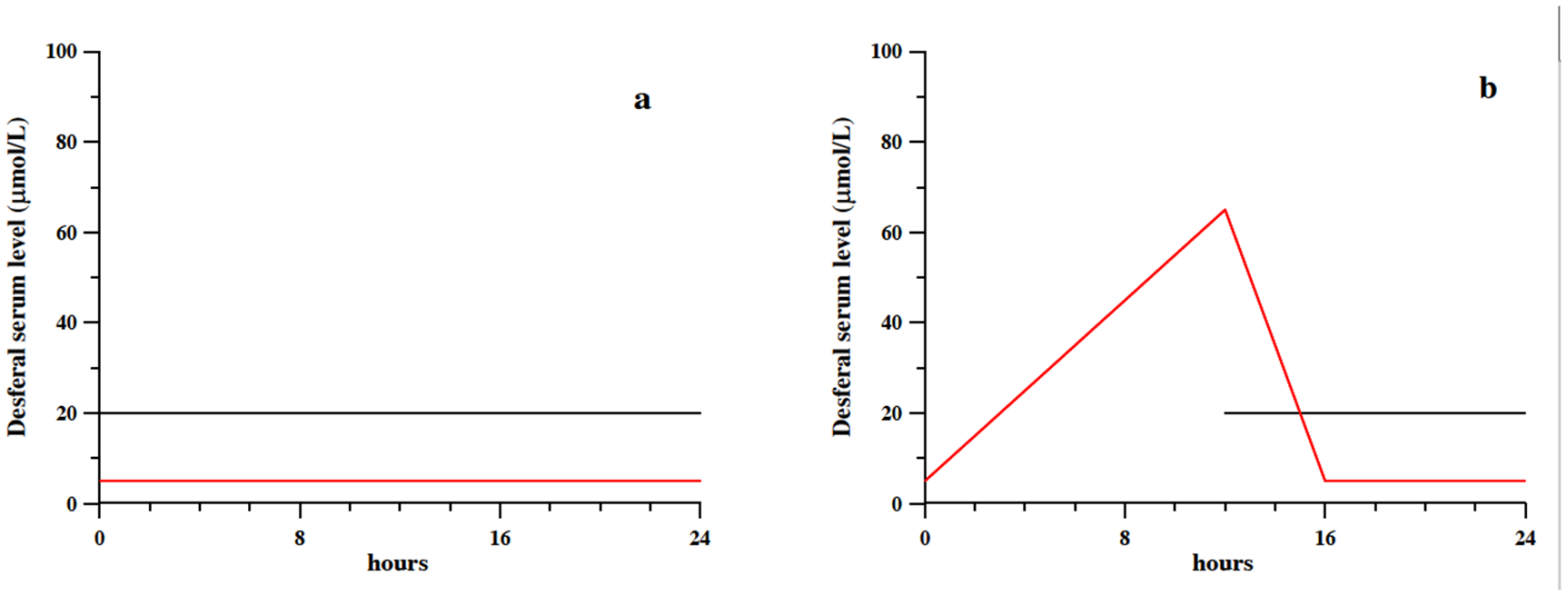
a) Plasma concentration of DFO (continuous line) reproduced on the basis of literature pharmacokinetic data [39] for a dose of 100 mg/Kg in 24 mL, administered at a rate of 1 mL per hour for 24 hours; the concentration of chelatable iron [25] is reported as red line. b) Plasma concentration of DFO (continuous line) for a dose of 100 mg/Kg in 12 mL, administered at a rate of 1 mL per hour for 12 hours. The concentration of chelatable iron [25], reported as red line, takes into account its increase when DFO is not administered.

An analysis of the pharmacokinetic behavior is necessary in order to correlate the amount of chelating agent available in plasma at a given time with the amount of drug administered, delivered in significantly greater amounts than the stoichiometric needs. For Desferal, Novartis reports that when a dose of 100 mg/Kg in 24 mL is administered at a rate of 1 mL per hour for 24 h, a plateau is reached with maximum levels of 20 μmol/L of ligand and 2.8 μmol/L of its iron complex in healthy subjects, and 8.3 μmol/L and 12.9 μmol/L in β-thalassaemia patients [39]. This allows us to infer a total circulating DFO concentration of about 22 μmol/L. Plasma concentration produced by a 100 mg/Kg dose administered in 24 hours corresponds to that produced by half a dose administered each 12 hours—i.e., the recommended dosage for transfusion-overload patient treatment. All of these considerations are illustrated in graphical form in Fig 5, where Fig 5a shows the iron (red line) and DFO (continuous line) concentrations in plasma (the DFO concentration necessary to stoichiometrically chelate iron in plasma is equal to that of iron). Fig 5a illustrates the ideal situation in which the chelating agent, continuously released for 24 h, acts on an always lower iron concentration, scavenging it and preventing its toxic action via the Fenton reaction. When the same dose of chelating agent is administered in 12 h, or less, in the hours between the end of the treatment and the beginning of the next one iron begins accumulating and reaches a concentration of about 65 μmol/L at the start of the new administration cycle. When the treatment starts all the administered DFO continuously chelates iron excess, and in about 4 hours a steady state situation is reached, which persists for the following 8 hours of treatment. Therefore, a 12-hour DFO administration protocol—illustrated in Fig 5b—implies that for almost 12 hours each day the patient is subject to dangerous concentrations of circulating iron. In such a case the prevalence of iron with respect to the chelating agent prevents any essential metal ion coordination by DFO.

#### DFP

**Fig 6:**
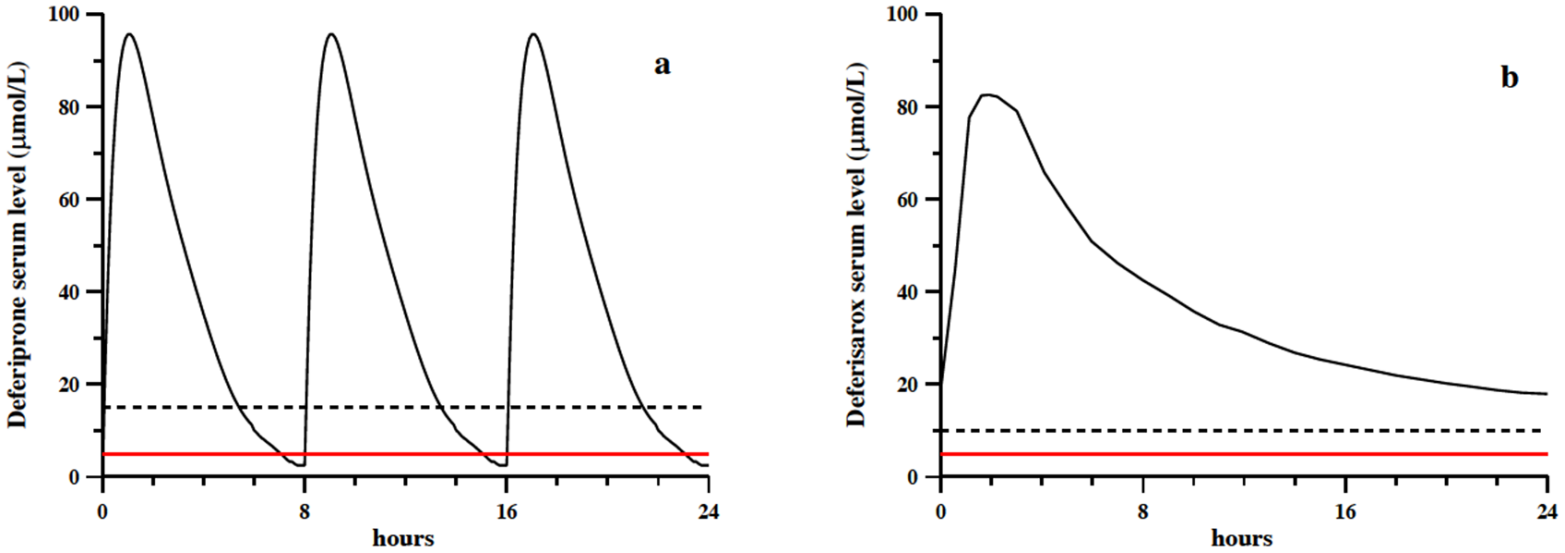
a) Plasma concentrations of DFP (continuous line) reproduced on the basis of literature pharmacokinetic data [41]; b) plasma concentrations of DFX (continuous line) reproduced on the basis of literature pharmacokinetic data [43]. In both cases a) and b) the concentration of chelatable iron [25] is reported as red line, and the stoichiometric ligand concentration needed to bind it as dashed line.

A pharmacokinetic study on DFP was performed by Thuma et al. [40] on five patients treated with an oral dose of 25 mg/Kg. The plasma DFP level in the five patients was measured for 8 hours and graphically reported in their paper as μg/mL *vs* time, allowing us to roughly evaluate the trend followed by the concentration of DFP in μmol/L (Fig 6a). For a standard patient assuming 75 mg/Kg/day in three doses, the trend repeats every 8 h. In Fig 6a the iron concentration in plasma (red line) and the DFP concentration necessary to stoichiometrically chelate iron in plasma (dashed line) are reported [41].

#### DFX

The pharmacokinetic trend reported in Fig 6b for DFX is based on the work by Nisbet-Brown et al. [42], relative to DFX administration at a dose of 20 mg/Kg/day; their works shows that the pharmacokinetic steady state trend is reached after 3 days of treatment [43]. In the same plot, the iron concentration in plasma is reported as red line, and the DFX concentration necessary to stoichiometrically chelate iron as dashed line.

## Results and Discussion

In this section we present the speciation of copper, zinc, and iron with the chelating agents in current use—DFO, DFP and DFX—to quantitatively evaluate the effects of iron(III) chelation on physiological concentration of essential metal ions copper(II) and zinc(II).

### Speciation at different simulated conditions

The pharmacokinetic profiles shown in Figs 5 and 6 helped us to choose a number of representative concentrations of ligands and metal ions to be used to calculate equilibrium points. For DFO three different situations were considered:

1. beginning of the treatment, at hour 12 in Fig 5b, characterized by a 20 μmol/L concentration of DFO and a 60 μmol/L iron concentration, therefore considering the non-chelated iron accumulated during the previous 12 hours without DFO administration;
2. three hours after starting the treatment, hour 15 in Fig 5b, with a 20 μmol/L concentration of both iron and DFO;
3. from hour 16 to hour 24 in both Fig 5a and in Fig 5b, corresponding to DFO and iron concentrations of 20 μmol/L and 5 μmol/L, respectively.

For all of these situations the concentrations of zinc and copper were considered to be 12 μmol/L and 2μmol/L, respectively.

On the other hand, for DFP, whose pharmacokinetic profile is depicted in Fig 6a, the following situations were considered:

1. from about 20 minutes after drug assumption and persisting for about two hours, characterized by a 70 μmol/L of DFP concentration and a 5 μmol/L iron concentration;
2. four hours after drug assumption, with concentrations of 35 μmol/L for DFP and 5 μmol/L for iron;
3. about 6 hours after drug assumption, characterized by a DFP concentration of 15 μmol/L and an iron concentration of 5 μmol/L;
4. the period between drug administration cycles, not depicted in Fig 6a, with a DFP concentration of 70 μmol/L (20 minutes after drug assumption) and an iron concentration of 20 μmol/L.

The same levels of zinc and copper as with DFO were assumed: 12 and 2 μmol/L respectively.

The choice of representative concentrations was simple in the case of DFX due to the single drug assumption. We considered three different situations:

1. the first four hours after drug assumption, corresponding to a DFX concentration of 80 μmol/L and an iron concentration of 5 μmol/L;
2. 4–8 hours after drug assumption, characterized by a 60 μmol/L and a 5 μmol/L concentration of DFX and iron, respectively;
3. a DFX concentration of 20 μmol/L, which persists for at least 16 hours until the following drug assumption, and an iron concentration level of 5 μmol/L.

A single iron concentration of 5 μmol/L was always assumed, being the DFX concentration always enough to chelate all circulating iron. The same levels of zinc and copper as in the cases of DFO and DFP were considered. Table 4 summarizes all the above choices.

**Table 4:** Speciation of different simulated conditions in the presence of the three different chelators. Total concentrations of ligands, iron, zinc and copper (μmol/L) in species calculations are reported in columns 2 to 5, while the concentrations (μmol/L) of calculated complexed species (disregarding minor species) are reported in the remaining columns.

Case	Chelator	Iron	Zinc	Copper	Iron-species		Zinc-species				Copper-species			
μmol/L					[FeLH]^+^	[FeL]	[ZnLH_2_]^+^	[ZnLH]	Zn_free_	Zn-Alb	[CuLH_2_]^+^	CuLH]	Cu_free_	Cu-Alb
DFO1	20	60	12	2	19.999	0.001	—	—	3.203	8.797	—	—	0.017	1.983
DFO2	20	20	12	2	19.999	0.001	—	—	3.203	8.797	—	—	0.017	1.983
DFO3	20	5	12	2	4.997	0.003	3.366	1.469	1.906	5.237	1.963	0.035	—	0.001
					[FeL_2_]^+^	[FeL_3_]	[ZnL]^+^	[ZnL_2_]	Zn_free_	Zn-Alb	[CuL]^+^	[CuL_2_]	Cu_free_	Cu-Alb
DFP1	70	5	12	2		4.544	4.998	1.526	1.592	4.338	—	1.972	—	—
DFP2	35	5	12	2		4.993	2.249	0.233	2.553	6.965	0.08	1.912	—	0.007
DFP3	15	5	12	2	0.547	4.453	0.034	—	3.212	8.756	0.226	0.064	0.014	1.696
DFP4	70	20	12	2	0.074	19.926	1.015	0.041	2.936	8.008	0.190	1.766	—	0.043
					[FeL_2_]^3-^		[ZnL]^-^		Zn_free_	Zn-Alb	[CuL]^-^		Cu_free_	Cu-Alb
DFX1	80	5	12	2	4.996		11.960		0.010	0.030	1.999		—	—
DFX2	60	5	12	2	4.996		11.935		0.017	0.048	1.999		—	—
DFX3	20	5	12	2	4.995		7.656		1.165	3.179	1.999		—	—

From the calculated concentrations reported in Table 4, disregarding the minor species, we can infer a number of observations:

- When the chelating agent is in excess with respect to iron (case DFO3), iron is completely chelated, copper is chelated for more than 99%, while zinc is chelated for about 40%. When iron concentration is higher or equal than that of DFO (cases DFO1 and DFO2), the chelating agent is completely involved in chelation with iron, and this prevents copper and zinc chelation. Iron is fundamentally found as [FeLH]^+^ whereas only negligible amounts of [FeL]^0^ are found. The only copper species is [CuLH_2_]^+^ while zinc is found as [ZnLH_2_]^+^ and [ZnLH]^0^ in comparable amounts.
- When DFP is in excess with respect to iron (DFP1, DFP2 and DFP4), all iron is chelated as the neutral [FeL_3_]^0^ complex, and copper is almost totally chelated, mainly as the [CuL_2_]^0^ complex. On the other hand, zinc is shared between chelator and albumin with a significant fraction of free metal ion. In particular, in case DFP1 zinc is bound to DFP for 51% as [ZnL]^+^ and [ZnL_2_]^0^, while the rest is either free (13%) or bound to albumin (36%). The chelated fraction decreases to 21% in case DFP2 and to 9% in case DFP4. When the amount of ligand that is necessary to stoichiometrically chelate iron as [FeL_3_]^0^, case DFP3, a particular situation is observed in which the presence of an essential metal ion perturbs iron chelation. Actually, iron, even if totally chelated, is found for 89% as [FeL_3_]^0^ and for 11% in the potentially dangerous [FeL_2_]^+^, since the ligand is also involved in copper chelation (11% of total copper as [CuL]).
- As far as DFX is concerned, it is always in excess with respect to chelatable iron, so the iron is totally complexed as [FeL_2_]^3-^; copper and zinc are also totally complexed, in the [ML]^-^ form, except in case DFX3 where 64% of zinc is found as [ZnL]^-^, 27% as Zn-albumin, and 9% as free ion.

The presence of the essential metal ions does not perturb iron chelation by DFO and DFX, while copper can interfere in iron chelation by DFP altering its distribution between [FeL_2_]^+^ and [FeL_3_]^0^. Such a condition is potentially dangerous since [FeL_2_]^+^ does not protect from iron participation in the Fenton reaction.

Within the limits of our assumptions, the present speciation study shows that iron chelation could imply the depletion or dislocation of essential metal ions, and that a thorough understanding of the copper and zinc complexation models is of paramount importance and must always be presented for any ligand intended to be used in therapy. Moreover, we would like to point out that iron chelators have been often proposed and used against aluminium toxicity [44]. The lower stability of aluminium complexes with respect to iron complexes is surely sufficient reason to expect a completely different picture of interference of essential metal ions that can both heavily affect aluminium chelation and give rise to their higher depletion. As such, a speciation study on aluminium chelation would be useful and difficult, given the paucity of data available for aluminium with respect to those existing for iron.

## Supporting Information

S1 Fig Structures of the three iron chelating agents currently in use. (TIFF)
